# The WMI Rat of Premature Cognitive Aging Presents Intrinsic Vulnerability to Oxidative Stress in Primary Neurons and Astrocytes Compared to Its Nearly Isogenic WLI Control

**DOI:** 10.3390/ijms25031692

**Published:** 2024-01-30

**Authors:** Adriana Ferreira, Aspen Harter, Sana Afreen, Karoly Kanai, Sandor Batori, Eva E. Redei

**Affiliations:** 1 Department of Cell and Developmental Biology, Feinberg School of Medicine, Northwestern University, Chicago, IL 60611, USA; a-ferreira@northwestern.edu (A.F.);; 2 Department of Psychiatry and Behavioral Sciences, Feinberg School of Medicine, Northwestern University, Chicago, IL 60611, USA; aspenharter2024@u.northwestern.edu; 3 Department of Organic Chemistry and Technology, Budapest University of Technology and Economics, 1111 Budapest, Hungary

**Keywords:** oxidative stress, mitochondrial dysfunction, gene expressions, strain differences

## Abstract

The primary neuronal and astrocyte culture described here is from the stress-hyperreactive Wistar Kyoto (WKY) More Immobile (WMI) rat with premature aging-related memory deficit, and its nearly isogenic control, the Less Immobile (WLI) strain. Primary WMI hippocampal neurons and cortical astrocytes are significantly more sensitive to oxidative stress (OS) generated by administration of H_2_O_2_ compared to WLI cells as measured by the trypan blue cell viability assay. Intrinsic genetic vulnerability is also suggested by the decreased gene expression in WMI neurons of catalase (*Cat*), and in WMI cortical astrocytes of insulin-like growth factor 2 (*Igf2*), synuclein gamma (*Sncg*) and glutathione peroxidase 2 (*Gpx2*) compared to WLI. The expressions of several mitochondrial genes are dramatically increased in response to H_2_O_2_ treatment in WLI, but not in WMI cortical astrocytes. We propose that the vulnerability of WMI neurons to OS is due to the genetic differences between the WLI and WMI. Furthermore, the upregulation of mitochondrial genes may be a compensatory response to the generation of free radicals by OS in the WLIs, and this mechanism is disturbed in the WMIs. Thus, this pilot study suggests intrinsic vulnerabilities in the WMI hippocampal neurons and cortical astrocytes, and affirm the efficacy of this bimodal *in vitro* screening system for finding novel drug targets to prevent oxidative damage in illnesses.

## 1. Introduction

Identifying factors that contribute to neurodegenerative processes in the brain can advance the treatments of these illnesses. The current mechanisms leading to the damage and death of brain cells in neurodegenerative diseases include oxidative stress (OS), which is caused by free radicals and mitochondrial alterations [1]. Chronic stress and depression are also known to increase OS and lead to changes in mitochondrial function [2]. Oxidative stress has a major role in the pathophysiology of disorders comorbid with depression [3,4]. The finding that advancing age is concomitant with increases in OS [5] generated the hypothesis that the accumulation of oxidative damage is the key driver of aging. This hypothesis implicated mitochondrial dysfunction in aging, as it is both the main source of ROS as it also detoxifies them. Oxidative stress may also be a key modulator and an early event in Alzheimer’s disease (AD), since the biomarkers of excessive oxidation appear early in its development [6,7,8].

Oxidative stress is a direct result of an imbalance between the production of reactive oxygen species (ROS), such as H_2_O_2_, and the availability of their specific antioxidant targets, such as superoxide dismutase (SOD), catalase (CAT) and glutathione peroxidase 1 and 2 (GPX1, GPX2) [1,8]. The expression of *Sod1* and *Cat* is decreased in the hippocampus of our early cognitive deficit model. This model, the Wistar Kyoto More Immobile (WMI) inbred rat strain, shows increased stress reactivity, enhanced depression-like behavior and deficits in learning and memory at 12 months of age, compared to its nearly isogenic control, the Wistar Kyoto Less Immobile strain (WLI) [9,10,11,12]. The WMI also exhibits decreased hippocampal expression of the learning and memory-promoting insulin-like growth factor 2 (*Igf2*) and its receptor (*Igf2r*) [9]. Thus, the WMI genetic differences from WLI predisposes them to age-related changes, including that of OS. Expressions of *SOD1*, *CAT*, *IGF2* and *IGF2R* also decreased in the postmortem brain regions of individuals with AD and depression compared those of AD, suggesting that the stress-related disorder of depression is indeed a risk factor for AD and exaggerates OS [13]. Thus, understanding ROS homeostasis would be important in the fight against neurodegenerative, and other diseases where OS is involved. For that purpose, an *in vitro* system that can predict vulnerability to OS would be beneficial.

We hypothesized that WLI and WMI would differ quantitatively in their cellular and molecular responses to oxidative stress and, ultimately, in their cell survival. The aim of this study was to determine whether these genetically unique, but nearly isogenic, strains would respond according to intrinsic individual patterns when subjected to oxidative challenges *in vitro* and whether such patterns could be determined and described. The *in vitro* systems explored were primary hippocampal neuronal and cortical astrocyte cultures from embryonic WLI and WMI. In addition to viability measures in response to OS, transcript levels of the genes that have shown aging-/depression-related changes in this model, as well as those relevant to mitochondrial dysfunction, were also determined. It is expected that the primary cultures of WMIs will be more sensitive to OS than those of WLIs, and therefore can be used in future studies for screening novel antioxidant targets. 

## 2. Results

### 2.1. Cell Viability in Response to H_2_O_2_

**Figure 1:**
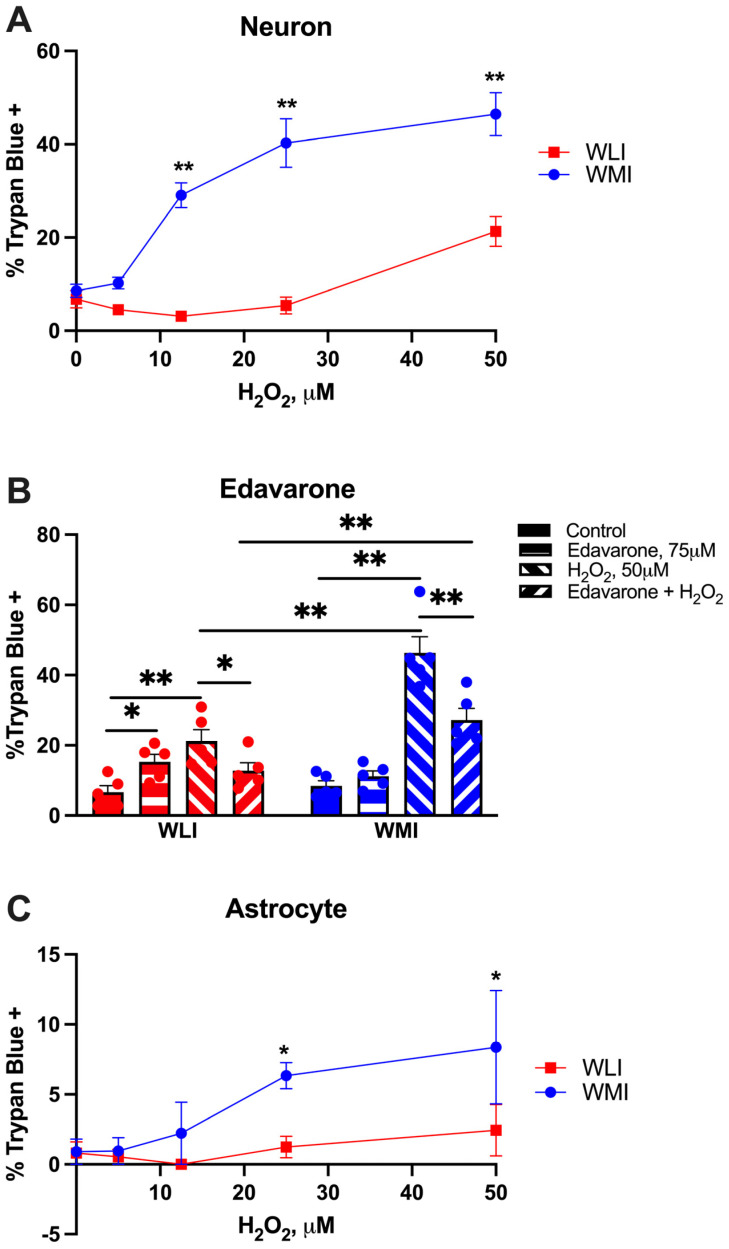
WMI hippocampal neurons and cortical astrocytes respond to increasing doses of H_2_O_2_ with significantly more cell death compared to WLI neurons and astrocytes. (**A**) The percentage of neurons with trypan blue inclusion [dead cells] was significantly higher in WMI neuronal cultures exposed to 12.5, 25 and 50 μM H_2_O_2_ than those of the WLI neurons. (**B**) Neuronal death induced by 50 μM H_2_O_2_ was reversable via the administration of 75 μM edavarone in both strains. (**C**) WMI cortical astrocytes showed a greater percentage of dead [trypan blue +] cells than WLIs at H_2_O_2_ doses of 25 and 50 μM. Values are mean +/− SEM from *n* = 5/strain/dose of H_2_O_2_ or other treatments. * *p* < 0.05, ** *p* < 0.01: comparison between WMI and WLI at the same dose of H_2_O_2_ for (**A**,**C**). Comparisons are as marked in (**B**).

Hippocampal WMI neurons were significantly more sensitive than WLI neurons to treatment with H_2_O_2_, responding with dramatically more cell death (strain, F[1,40] = 112.3, *p* < 0.001; dose of H_2_O_2_, F[4,40] = 31.97, *p* < 0.001; strain × dose, F[4,40] = 12.96, *p* < 0.001; Figure 1A). The H_2_O_2_ dose response illustrates that WMI neurons responded to 10 μM H_2_O_2_, with a similar level of trypan blue uptake in WLI neurons after 50 μM H_2_O_2_. The cell viability seemed to be specific to the effect of oxidative stress generated by H_2_O_2_, as administration of the antioxidant Edavarone (75 μM) significantly decreased the cell death induced by 50 μM of H_2_O_2_ in the neurons of both strains (strain, F[1,32] = 23.09, *p* < 0.001; H_2_O_2_ treatment, F[1,32] = 72.20, *p* < 0.001; Edavarone treatment F[1,32] = 4.34, *p* = 0.045; strain × H_2_O_2_, F[1,32] = 29.09, *p* < 0.001; strain × Edavarone, F[1,32] = 4.60, *p* = 0.04, H_2_O_2_ × Edavarone, F[1,32] = 25.22, *p* < 0.001; Figure 1B).

Cortical astrocytes were not as sensitive to oxidative stress as hippocampal neurons, although WMI astrocytes responded with significantly greater cell death compared to WLIs at higher H_2_O_2_ concentrations (strain, F[1,40] = 6.55, *p* = 0.014; H_2_O_2_ dose, F[4,40] = 3.07, *p* = 0.027; Figure 1C).

### 2.2. Neuron-Specific Gene Expression Responses to OS

**Figure 2:**
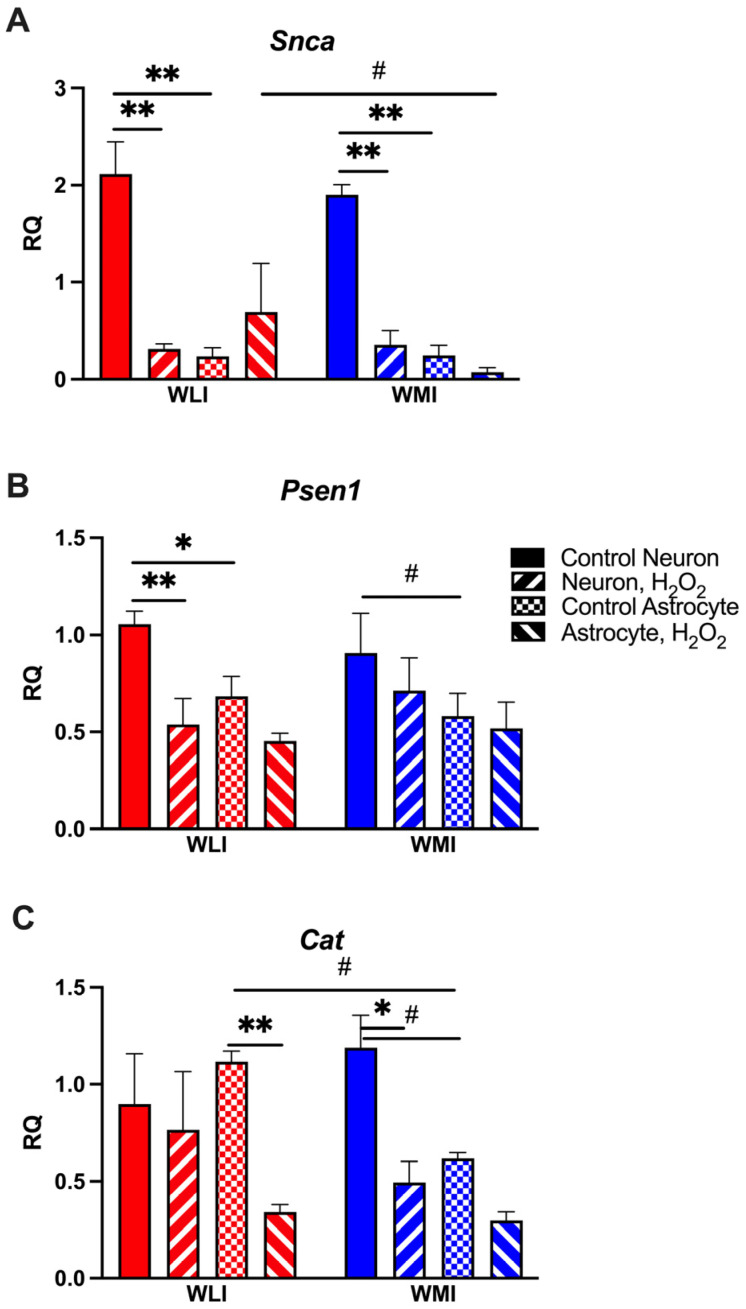
Neuronal gene expression differences between WLI and WMI in response to 50 μM of H_2_O_2_. (**A**) Synuclein alpha (*Snca*) expression is significantly greater in neurons than in astrocytes, and this neuronal expression is decreased in response to H_2_O_2_. (**B**) Presenilin 1 (*Psen1*) expression is also significantly greater in neurons than in astrocytes, particularly in WLIs, and it is reduced by H_2_O_2_ in WLI, but not in WMI neurons. (**C**) Catalase (*Cat*) expression is reduced by H_2_O_2_ in WMI neurons only, while only WLI astrocytes respond with significantly reduced transcript levels to the same treatment. Note that WMI astrocytes tend to express less *Cat* than WLIs. Values are mean +/− SEM from *n* = 3–4/strain/cell type/treatment. * *p* < 0.05, ** *p* < 0.01, # *p* < 0.1; comparisons are as marked.

Synuclein alpha (*Snca*), *Psen1* and *Cat* transcript levels have decreased in response to OS induced by H_2_O_2_ in either or both strains’ neuronal culture (Figure 2). The *Snca* and *Psen1* expressions were significantly higher in hippocampal neurons compared to cortical astrocytes in both strains. H_2_O_2_ treatment reduced *Snca* expression significantly in both strains, while only the WLI neurons responded with reduced *Psen1* levels (*Snca*: cell type, F[1,15] = 25.55, *p* < 0.001; H_2_O_2_, F[1,15] = 20.37, *p* < 0.001; cell type × H_2_O_2_, F[1,15] = 28.83, *p* < 0.001; Figure 2A; *Psen1:* cell type, F[1,17] = 7.34, *p* = 0.015; H_2_O_2_, F[1,17] = 7.80, *p* = 0.013; Figure 2B). In contrast, WMI hippocampal neurons, but not WLI, showed a trend of greater expression of the antioxidant *Cat* compared to cortical astrocytes, and a significantly decreased expression after treatment with H_2_O_2_ (cell type, F[1,16] = 3.65, *p* = 0.074; H_2_O_2_, F[1,16] = 14.26, *p* < 0.01; Figure 2C). Catalase transcript levels decreased in WLI, but not WMI, astrocytes in response to H_2_O_2_ (strain × cell type × H_2_O_2_, F[1,16] = 3.98, *p* = 0.064).

### 2.3. Astrocyte-Specific Gene Expression Responses to OS

**Figure 3:**
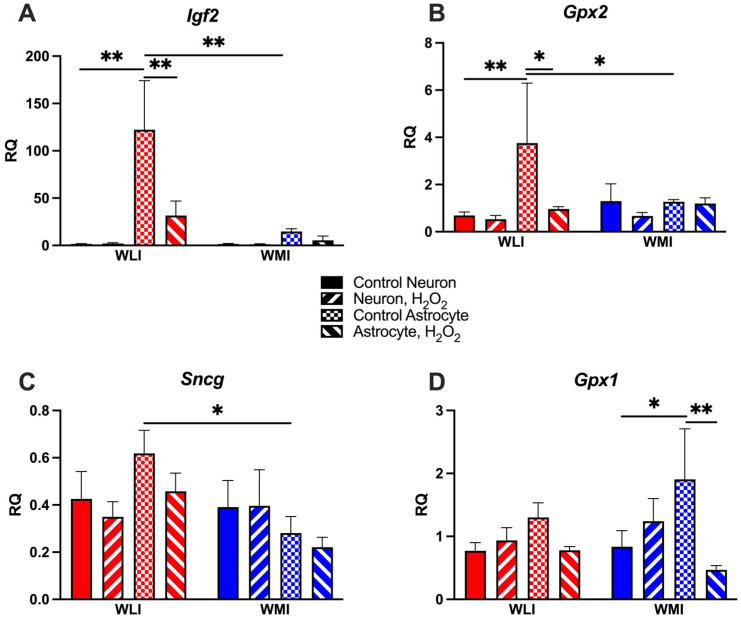
Gene expression differences in cortical astrocytes between WLI and WMI in response to 50 μM of H_2_O_2_. (**A**) Insulin-like growth factor 2 (*Igf2*) expression is significantly greater in WLI than WMI astrocytes, and this expression decreased in response to H_2_O_2_ in the WLI. (**B**) Glutathione peroxidase 2 (*Gpx2*) expression is also significantly greater in WLI astrocytes than in WLI neurons, and also compared to WMI astrocytes. The expression is reduced in response to H_2_O_2_ in WLI astrocytes. (**C**) Transcript levels of synuclein gamma (*Sncg*) are greater in WLI than WMI astrocytes. (**D**) Gutathione peroxidase 1 (*Gpx1*) expression shows a similar pattern by cell type and treatment in both WLIs and WMIs, although the differences are more pronounced in WMIs. Values are mean +/− SEM from *n* = 3–4/strain/cell type/treatment. * *p* < 0.05, ** *p* < 0.01, comparisons are as marked.

*Igf2*, *Gpx2* and *Sncg* expressions were significantly greater in WLI cortical astrocytes compared to WMIs’ (Figure 3). Subsequently, H_2_O_2_ treatment only reduced *Igf2* and *Gpx2* transcript levels in WLI astrocytes (***Igf2***: strain, F[1,17] = 6.75, *p* = 0.019; cell type, F[1,17] = 10.48, *p* = 0.005; H_2_O_2_, F[1,17] = 3.73, *p* = 0.070; strain × cell type, F[1,17] = 6.60, *p* = 0.020; cell type × H_2_O_2_, F[1,17] = 3.76, *p* = 0.069; Figure 3A; ***Gpx2****:* cell type, F[1,15] = 4.81, *p* = 0.045; H_2_O_2_, F[1,15] = 3.99, *p* = 0.064; Figure 3B). *Sncg* expression differed between WLI and WMI astrocytes without any treatment (strain, F[1,17] = 3.88, *p* = 0.066; strain × cell type, F[1,17] = 4.23, *p* = 0.055; Figure 3C).

The expression of *Gpx1* was greater in astrocytes than in neurons, particularly in WMIs (Figure 3D). This increased expression was significantly reduced by H_2_O_2_ treatment (cell type × H_2_O_2_, F[1.17] = 7.22, *p* = 0.016).

Transcript levels of *Igf2r* and *Sod1* were not changed in response to H_2_O_2_ treatment. Interestingly, *Igf2r* expression was significantly lower in WMI astrocytes than in WMI neurons, while there were no cell-type differences in WLIs (*Igf2r*: cell type, F[1,17] = 18.24, *p* < 0.001; strain × cell type, F[1,17] = 4.65, *p* = 0.046; *Sod1*: No significant results; Supplemental Figure S1).

### 2.4. Mitochondrial Gene Expression

**Figure 4:**
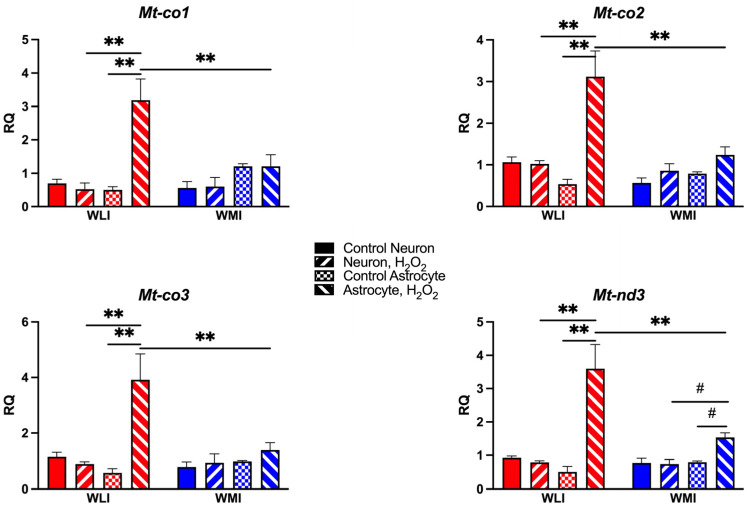
Transcript levels of mitochondrial genes significantly increased in response to 50 μM H_2_O_2_ only in WLI cortical astrocytes. Expression of all four mitochondrial genes increased after treatment with H_2_O_2_ in WLI astrocytes, but not in WLI and WMI neurons and WMI astrocytes. The WMI *Mt-nd3* expression tended to follow the WLI pattern. Values are mean +/− SEM from *n* = 3–4/strain/cell type/treatment. ** *p* < 0.01, # *p* < 0.1, comparisons are as marked.

Based on expression differences between middle-aged WLI and WMI hippocampal RNA-seq, transcript levels of selected mitochondrial genes were measured in both hippocampal neuronal and cortical astrocyte cultures. Expression profiles of *Mt-co1*, *Mt-co2*, *Mt-co3*, and *Mt-nd3* were very similar (Figure 4). WLI cortical astrocytes expressed exaggerated responses to H_2_O_2_, while the WLI and WMI hippocampal neurons, and most importantly the WMI astrocytes, were mostly unresponsive to H_2_O_2_ treatment. The three-way ANOVA results for these transcript level changes are as follows: ***Mt-co1***: cell type, F[1,17] = 21.15, *p* < 0.001; H_2_O_2_, F[1,17] = 9.93, *p* = 0.006; strain × H_2_O_2_, F[1,17] = 9.29, *p* = 0.007; cell type × H_2_O_2_, F[1,17] = 12.17, *p* = 0.003; strain × cell type × H_2_O_2_, F[1,17] = 12.88, *p* = 0.002; ***Mt-co2***: strain, F[1,17] = 11.03, *p* = 0.004; cell type, F[1,17] = 10.06, *p* = 0.006; H_2_O_2_, F[1,17] = 22.79, *p* < 0.001; strain × H_2_O_2_, F[1,17] = 6.93, *p* = 0.018; cell type × H_2_O_2_, F[1,17] = 16.39, *p* < 0.001; strain × cell type × H_2_O_2_, F[1,17] = 12.97, *p* = 0.002; ***Mt-co3***: strain, F[1,17] = 5.65, *p* = 0.030; cell type, F[1,17] = 9.21, *p* = 0.008; H_2_O_2_, F[1,17] = 12.55, *p* = 0.003; strain × H_2_O_2_, F[1,17] = 6.10, *p* = 0.024; cell type × H_2_O_2_, F[1,17] = 14.07, *p* = 0.002; strain × cell type × H_2_O_2_, F[1,17] = 10.59, *p* = 0.005; ***Mt-nd3***: strain, F[1,17] = 6.88, *p* = 0.018; cell type, F[1,17] = 18.27, *p* < 0.001; H_2_O_2_, F[1,17] = 23.87, *p* < 0.001; strain × H_2_O_2_, F[1,17] = 9.02, *p* = 0.008; cell type × H_2_O_2_, F[1,17] = 28.39, *p* < 0.001; strain × cell type × H_2_O_2_, F[1,17] = 10.63, *p* = 0.005.

## 3. Discussion

The findings of this study suggest that genetic differences between the nearly isogenic strains of WLI and WMI affect their responses to oxidative stress *in vitro*. Isolated neurons and astrocytes were employed due to the neurons’ particular vulnerability to oxidative stress, and the low expression of antioxidant levels in neurons compared to astrocytes [14]. Accordingly, neuronal death was greater than astrocyte death in response to H_2_O_2_, and WMI cells were dramatically more sensitive than WLIs. Additionally, the findings suggest intrinsic vulnerabilities in the WMI astrocytes, including decreased *Cat*, *Igf2*, *Gpx2*, *Sncg* and mitochondrial complex 4 expressions compared to the WLI cortical astrocytes.

There are limited data on genetic differences in response to oxidative stress. Gunther et al. [15] describes different types of oxidative stressors resulting in phenotypic differences in isolated neurons from two genetically distinct strains of rats. They argue that their findings should be considered in future individualized treatments and when evaluating antioxidative pharmacological interventions. The increased sensitivity of WMI to OS *in vitro* is of particular interest since middle-aged WMIs (12 months old) show premature cognitive decline compared to same age WLIs and their young (6 months old) counterparts [9,16]. We propose that the intrinsic vulnerability of WMI to oxidative stress could contribute to their premature cognitive decline.

The inbred WMI strain is a genetic model of depression [12]. Since major depression is a risk factor for the development of dementia [17,18], there is a proof of concept in this connection in that the depression model WMI also shows premature cognitive deficit. Interestingly, recent studies suggest that antidepressant treatments can ameliorate oxidative stress and Aβ-induced toxicity in an animal model of AD, which could lead to attenuating cognitive deficits and depressive-like phenotypes [19]. The prevalence of both major depressive disorder (MDD) [20,21] and dementia [22,23] are higher in women than age-matched men. Both the progression of cognitive decline and its association with MDD shows that females are particularly vulnerable [24,25]. Higher risks of cognitive decline and dementia have been associated with the menopause before the age of 40–45, representing a female-specific risk factor for AD dementia [26]. Appropriately, WMI females show a greater cognitive deficit in middle age compared to the WMI males or younger males and females of both strains [9,16], suggesting further appropriateness of the model.

These gender differences are explained by differences in sex hormones and their decline with age [27,28]. Estrogen can act as an antioxidant, therefore the declining estrogen levels with aging or surgery could lead to amplified oxidative stress processes [29,30]. Whether there is a role in sex differences in the intrinsic vulnerability of WMI to OS is difficult to determine. Our studies were performed using neuronal and astrocyte cultures prepared from both female and male embryos together; therefore if sex-related differences exist in the susceptibility differences between WMI and WLI to oxidative stress, it could not be detected. To our knowledge, no one has measured estrogen levels in primary neuronal or astrocyte cultures generated from E18 brain regions. Therefore, this question remains open unless neuronal and astrocyte cultures, prepared from only female or male WMI and WLI embryos, are exposed to oxidative stress and their responses are studied specifically.

Despite the increased oxidative stress response of WMI cultures as compared to the WLI cultures, edaravone treatment significantly reduced neuronal susceptibility to hydrogen peroxide in both strains. Edaravone modulates oxidative damage in various diseases, including neurodegenerative diseases. It is also suggested that it can restore mitochondrial dysfunction [31,32,33]. Alternatively, edaravone could induce neuroprotective effects by decreasing NF-κB, proinflammatory cytokines and inflammatory protein levels in the hippocampus [34]. Further studies could clarify whether edavarone and other antioxidants affect the cortical astrocyte response and the mitochondrial gene expression to hydrogen peroxide treatment in WMI and WLI. Future *in vivo* studies aiming to prevent premature cognitive decline in middle-aged WMIs with antioxidant treatments would be very informative, particularly in light of the increased attention in preventing AD [35].

Hippocampal neurons were chosen for the primary neuronal culture as this brain region is particularly important for cognition and it is also known to be specifically affected by OS [36]. Additionally, cortical astrocytes were investigated as accumulating evidence indicates their protective role against oxidative stress. One argument is that astrocytes exhibit a protective nuclear factor erythroid 2-related factor 2 (*Nrf2*) response that regulates the expression of antioxidants and thereby protects against oxidative damage. Considering the weak activity of the neuronal Nrf2 pathway, enhancing glial rather than neuronal antioxidant capacity can better modulate CNS antioxidant defenses [37]. In agreement with this, hippocampal neurons in general were more vulnerable to H_2_O_2_ treatment-induced cell death than cortical astrocytes in the present study. The significantly higher sensitivity of WMI cortical astrocytes to this OS compared to WLIs further suggests that WMI astrocytes are less capable providing protection against oxidative damage than WLIs, thereby expanding the intrinsic vulnerability of WMIs to OS.

The transcript levels of the antioxidant enzymes *Cat* and *Gpx2* were also lower in WMI astrocytes, even before treatment with H_2_O_2_. Lower hippocampal *Cat* expression is shown in middle-aged WMIs compared to same-age WLIs, as well as in the human hippocampus and anterior cingulate cortex of subjects with both depression and Alzheimer’s Disease [9,13]. As both aging and depression are known to show increased ROS, the innate reduction of *Cat* expression can contribute to the vulnerability of WMIs to aging-induced elevated ROS. The assessment of the decreased *Gpx2* expression in the WMI cortical astrocytes is not simple, as *Gpx2* is thought to be primarily expressed in the gastrointestinal system [38]. Nevertheless, Gpx2 is one of the major defenses against oxidative stress, and the current findings may shed light on how it can be present and regulated in the embryonic brain.

The dramatically lower expression of *Igf2* in the WMI cortical glia compared to WLIs is an additional argument for the applicability of this *in vitro* system to find novel drug targets. The expression of the learning and memory-stimulant *Igf2* is lower in the middle-aged WMI hippocampus compared to WLI and WMI hippocampi of young animals [9]. Furthermore, attenuated levels of hippocampal IGF2 have also been proposed to contribute to AD pathology [39]. Therefore, should middle-aged cortical astrocytes of WMI express lower levels of *Igf2* in addition to the whole hippocampus, they could contribute to the premature cognitive decline present in the WMIs.

The intrinsic vulnerability of WMIs to OS could be related to the absence of increased expression in response to H_2_O_2_ treatment in WMI astrocytes, in contrast to the large increases in WLIs. An increase in Mt-co1 has been observed previously in the lymph nodes of older mice compared to younger mice, and the suggestion that these increases are compensatory to an OS challenge was made [40]. The complex I, or nicotine-amide adenine dinucleotide (NADH) dehydrogenase, of which Mt-nd3 is part of, is the largest protein complex in the mitochondrial respiratory chain, and it also showed a decreased expression in H_2_O_2_-stimulated WMI astrocytes compared to WLIs. It can be hypothesized that the lack of increases in the mitochondrially encoded subunits of the respiratory chain complexes in WMIs can have a main role in premature cognitive aging of these animals. Mitochondrion is an important organelle in the eukaryotic cells, which acts as a major player in energy metabolism and ATP production [41]. Therefore, the dysfunction of the WMI astrocyte might lead to the disorder of the mitochondrial respiratory chain and the decline of ATP production in WMIs [42].

These experiments were performed using well characterized primary hippocampal neuronal and cortical astrocyte cultures that provided homogenous cell populations. We acknowledge the reductionist nature of this experimental approach, and therefore, the data obtained should be interpreted with this caveat in mind. Additionally, hippocampal astrocytes and cortical neurons should be studied in the future, together with hippocampal neurons and cortical astrocytes, to ascertain the role of the different cell types in their immediate environment. Since we assessed changes in the expression of a limited number of genes associated with oxidative stress responses, future transcriptomic studies will identify genes involved in the susceptibility differences in neurons and astrocytes to oxidative stress between WMI and WLI rats.

Taken together, the present study suggests that WMI hippocampal neurons and cortical astrocytes have an intrinsic vulnerability to oxidative stress compared to their near-isogenic counterparts, the WLIs. This intrinsic vulnerability occurs in the absence of any environmental effects, and perhaps forms the basis for the aging-induced premature cognitive decline observed in middle-aged WMIs. Since the genetic difference between these two strains is limited to a low number of approximately 7000 sequence variations [11], *in vitro* screening using these strains could identify potential drug targets for cognitive dysfunction.

## 4. Materials and Methods

### 4.1. Animals

The animals were maintained at Northwestern University Feinberg School of Medicine satellite facility adjacent to Dr. Redei’s laboratory. All animal procedures were approved by the Institutional Animal Care and Use Committee of Northwestern University (IS00001385, 2015; IS00021232, 2022). The Wistar Kyoto Less Immobile(WLI/Eer; WLI) and Wistar Kyoto More Immobile (WMI/Eer; WMI) inbred strains were housed under temperature and humidity control with food and water *ad libitum* on a 12:12 LD cycle with lights on at 0600 h. Females of both strains were mated for this study.

### 4.2. Hippocampal Culture Preparation

Embryonic day E18 WLI and WMI rat embryos were used for the preparation of the hippocampal cultures as previously described [43,44]. In brief, hippocampi were dissected, stripped of meninges, trypsinized (0.25%) for 15 min at 37 °C, and dissociated by pipetting gently through a fire-polished Pasteur pipette. Neurons were plated on poly-L-lysine-coated coverslips (250,000 cells/60 mm dish) in a Minimum Essential Medium (MEM, Thermo Fisher Scientific, Waltham, MA, USA) containing 10% horse serum (MEM10, Thermo Fisher Scientific, Waltham, MA USA) for cell survival assays. Neurons were plated at high density (1,000,000 cells/60 mm dish) on poly-L-lysine-coated dishes for RNA extraction and gene expression analysis. After 4h, the medium was replaced with an astrocyte-conditioned MEM containing N2 supplements, ovalbumin 0.1%- and 0.1-mM sodium pyruvate [N2 medium; 44, 45]. Neurons were cultured for seven days.

### 4.3. Preparation of Astrocyte Cultures

The astrocyte cultures were prepared from the cerebral cortex of E18 WLI and WMI rat embryos as previously described [45,46]. Briefly, embryos were removed, and their cerebral cortex dissected and freed of meninges. The cells were dissociated by trypsinization (0.25% for 35 min at 37 °C) and then centrifuged in MEM10 medium at 500× *g* for 10 min. The cells were resuspended in a fresh MEM10 medium, triturated with a fire-polished pipette, and plated at low (250,000 cells/60 mm dish) and high density (1,000,000 cells/60 mm dish) on non-coated culture dishes for cell survival assay and RNA isolation, respectively. Astrocytes were cultured in MEM10 for seven days.

### 4.4. Preparation of N2 Astrocyte-Conditioned Medium

To prepare the N2 astrocyte-conditioned medium, astrocytes were isolated from the cerebral cortex of E18 Sprague Dawley rat embryos as described above. Dissociated astrocytes were plated at high density (2,000,000 cells/75 mL flaks) and cultured in MEM10. Once they reached confluence, the medium was replaced with an N2 medium. The N2-astrocyte-conditioned medium was collected 24 h later [44,45].

### 4.5. Drug Treatments

Hydrogen peroxide (H_2_O_2_] (Sigma Aldrich, Burlington, MA, USA) and the antioxidant edaravone [3-Methyl-1-phenyl-5-pyrazolone] (Thermo Fisher Scientific, Waltham, MA, USA) were diluted in MilliQ sterile water to obtain stock solutions of 9.8 mM and 7.5 mM, respectively. Reagents were added to seven-day cultured neurons and astrocytes as described [47,48,49,50].

For dose-response studies measuring cell survival, cultured neurons or astrocytes were divided into five groups: the control group, where the cells received no drug treatment; and the H_2_O_2_ groups, where the cells were incubated with 5, 12.5, 25 or 50 μM H_2_O_2_ for 24 h.

The cultured neurons were divided into four groups in the edaravone experiment: [1] the control group, where the cells received no drug treatment; [2] the edaravone group, where the cells were incubated with 75 μM of edaravone for 24 h; [3] the H_2_O_2_ group, where the cells were incubated with H_2_O_2_ (50 μM) for 24 h; and [4] the edaravone plus H_2_O_2_ group, where the cells were incubated with 75 μM of edaravone for 24 h prior to their incubation with 50 μM of H_2_O_2_ for an additional 24 h.

For gene expression studies, neurons and astrocytes were cultured either without any treatment or with 50 μM H_2_O_2_ for 24 h.

### 4.6. Determination of Cell Survival

To determine neuronal and astrocyte survival, at the end of the experiments, the media were removed and replaced with 1 mL of 1:1 dilution of trypan blue (0.4% Sigma Aldrich, Burlington, MA, USA) in PBS. The cells were incubated for 10 min, washed twice with 1 mL PBS, and counted in 5 fields at 20× magnification under a bright field microscope. The percentage of dead cells was determined as the percentage of trypan blue positive cells in the number of total cells [50].

### 4.7. RNA Extraction and Rreverse Transcription

Total RNA was extracted using the TRIzol Reagent (Sigma Aldrich, Burlington, MA, USA) per the manufacturer’s protocol. Briefly, neuronal and astrocyte cultures were homogenized with RNase-free Teflon pestles and glass tissue homogenizers. The total RNA was extracted with TRizol and chloroform (0.2 mL), precipitated with 0.5 mL of isopropanol. RNA samples for the Real-Time Reverse Transcription-Polymerase Chain Reaction were quantified using a Nanodrop Spectrophotometer (Nanodrop Technologies, Wilmington, DE, USA).

Once isolated, 1 µg of the total RNA was reverse transcribed using the SuperScript VILO cDNA Synthesis Kit (Thermo Fisher Scientific, Waltham, MA, USA). All of these methods have been described previously [9,51].

### 4.8. Real-Time Reverse Transcription-Polymerase Chain Reaction [RT-qPCR]

For each experimental group, RT-qPCR was performed to compare the neuron or astrocyte target gene expression levels between strains (WLI vs. WMI) and treatments. Primers for each target gene were designed using Applied Biosystems Primer Express software (version 3.0, Applied Biosystems, Foster City, CA, USA); the primer sequences can be found in Supplemental Table S1. Five ng of cDNA were amplified in a 20µL reaction using qPCR 2X MasterMix Universal for SYBR Green Assay [Lamda Biotech, St. Luis, MO, USA] in the QuantStudio 6 Flex Real-Time PCR System [Thermo Fisher Scientific, Waltham, MA, USA]. Triplicates of the reactions were performed and reached threshold amplification within 34 cycles. The target transcript levels were quantified relative to *Gapdh*, a housekeeping gene previously demonstrated to show similar expression across strains and conditions, and to a WLI control neuronal cDNA calibrator using the 2^−[∆∆CT]^ method.

### 4.9. Statistical Analysis

Quantitative analysis and statistical comparisons were performed using Prism 10.1.0 [GraphPad Software, Inc., San Diego, CA, USA]. The neuron and astrocyte survival data were compared between the strains across the H_2_O_2_ dose response using two-way ANOVA [strain, dose of H_2_O_2_] followed by a Fisher’s LSD *post hoc* test. The edavarone treatment data and all gene expression data were analyzed by three-way ANOVA [strain, cell type and treatment] followed by a Fisher’s LSD *post hoc* test. Values of *p* < 0.05 were considered significant. Data are presented as mean ± standard error of the mean [S.E.M.] and post-hoc statistical significance were indicated in the graphs.

## Data Availability

Raw data supporting the findings described in this work will be made available upon request.

## Supplementary Materials

The following supporting information can be downloaded at: https://www.mdpi.com/article/10.3390/ijms25031692/s1.
