# Supplementary material for: The WMI Rat of Premature Cognitive Aging Presents Intrinsic Vulnerability to Oxidative Stress in Primary Neurons and Astrocytes Compared to Its Nearly Isogenic WLI Control
Source: Int J Mol Sci. 2024 Jan 30;25(3):1692. doi: 10.3390/ijms25031692 (PMC10855588; doi:10.3390/ijms25031692)
Supplement: Supplementary file 1 [file ijms-25-01692-s001.zip › ijms-2823383-supplementary.pdf]

Supplemental Material:

The WMI Rat of Premature Cognitive Aging Presents Intrinsic Vulnerability to Oxidative Stress in Primary Neurons and Astrocytes Compared to Its Nearly Isogenic WLI Control

Adriana Ferreira [1], Aspen Harter [2], Sana Afreen [1], Karoly Kanai [3], Sandor Batori [3] and Eva E Redei [2,*]

[1] Department of Cell and Developmental Biology, Feinberg School of Medicine, Northwestern University, Chicago, IL 60611, USA; a-ferreira@northwestern.edu (A.F.); sana.afreen@law.northwestern.edu (S.A.)
[2] Department of Psychiatry and Behavioral Sciences, Feinberg School of Medicine, Northwestern University, Chicago, IL 60611, USA; aspenharter2024@u.northwestern.edu
[3] Department of Organic Chemistry and Technology, Budapest University of Technology and Economics, Budapest, 1111, Hungary; kkanai@edu.bme.hu (K.K.); batori.sandor@edu.bme.hu (S.B.)
* Correspondence: e-redei@northwestern.edu; Tel.: +1-(312)-908-1791

Supplemental Table 1. Primer sequences for quantitative RT-PCR

**Gene**		**Sequence 5'-3'**
Igf2	*F*	TCA GCA AGT GCC TAA AGA ATG G
	R	TGG GTG GAT GTC CAG TCA AA
Igf2r	*F*	CAG TGG GTG ACT CAC TTT TGA GAA
	R	GCC CGT AGT GGT GTT AAA TTC C
Cat	*F*	CAG GGA TGC GAT GTT GTT TCC
	R	AAG GTG TGT GAG CCA TAG CC
Sod1	*F*	CAG AAG GCA AGC GGT GAA C
	R	CCA TGC TCG CCT TCA GTT AAT C
Gpx1	*F*	TCA GTT CGG ACA TCA GGA GAA T
	R	TCA CCA TTC ACC TCG CAC TT
Gpx2	*F*	TCG CTT ACA TCG CCA AGT CT
	R	CCT CAT TCT GAC AGT TCT CCT GA
Psen1	*F*	TTG ATC GGC CTG TGC CTT A
	R	CCG GCA ACG CCT TCT TGA
Snca	*F*	GAT GGG CAA GGG TGA AGA AG
	R	GCT AGG GTC CAC AGG CAT GT
Sncg	*F*	AAG TGT AAC CTC GGT GGC TG
	R	TCT TGC TCT TTG GCC TCC TG
Mt-co1	*F*	ATT GGA GGC TTC GGG AAC TG
	R	AGA TAG AAG ACA CCC CGG CT
Mt-co2	*F*	TGG CTT ACA AGA CGC CAC AT
	R	TGG GCG TCT ATT GTG CTT GT
Mt-co3	*F*	AAG GCC ACC ACA CCC CTA TT
	R	TAA TTC CTG TTG GGG GTC AGC
Mt-nd3	*F*	GAC CAA CAA GTT CTG CAC GC
	R	AGG GGG AGT AGT AAG GCG AT
Gapdh	*F*	CAA CTC CCT CAA GAT TGT CAG CAA
	R	GGC ATG GAC TGT GGT CAT GA

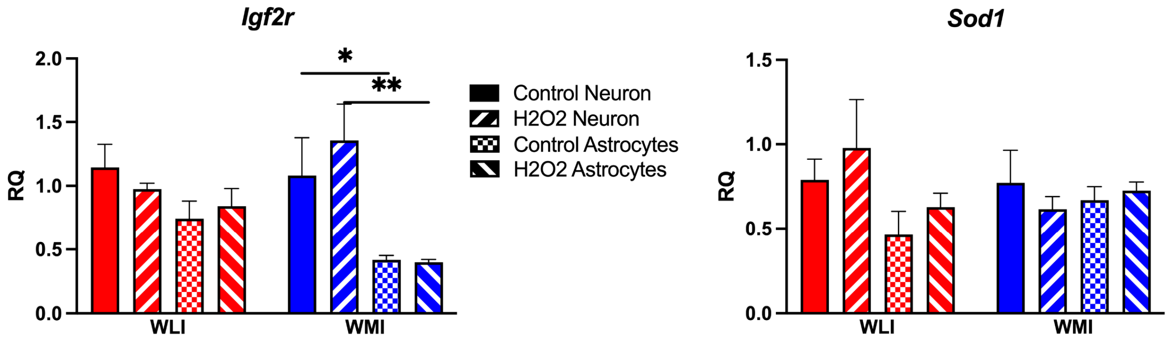

Supplemental Figure 1. ***Igf2r*** **and** ***Sod*** **1 transcript levels did not change in response to 50mM H_2O_2 treatment.** WMI hippocampal neurons expressed greater levels of *Igf2r* compared to WMI astrocytes. Values are mean +/- SEM from n=3-4/strain/cell type/treatment. *p<0.05, **p<0.01, comparisons as marked.
